# Low Temperature Thermal Atomic Layer Deposition of Aluminum Nitride Using Hydrazine as the Nitrogen Source

**DOI:** 10.3390/ma13153387

**Published:** 2020-07-31

**Authors:** Yong Chan Jung, Su Min Hwang, Dan N. Le, Aswin L. N. Kondusamy, Jaidah Mohan, Sang Woo Kim, Jin Hyun Kim, Antonio T. Lucero, Arul Ravichandran, Harrison Sejoon Kim, Si Joon Kim, Rino Choi, Jinho Ahn, Daniel Alvarez, Jeff Spiegelman, Jiyoung Kim

**Affiliations:** 1 Department of Materials Science and Engineering, The University of Texas at Dallas, 800 West Campbell Road, Richardson, TX 75080, USA; Yongchan.Jung@utdallas.edu (Y.C.J.); SuMin.Hwang@utdallas.edu (S.M.H.); Dan.Le@utdallas.edu (D.N.L.); aswinlakshminarayanan.kondusamy@utdallas.edu (A.L.N.K.); Jaidah.Mohan@utdallas.edu (J.M.); SangWoo.Kim@utdallas.edu (S.W.K.); Jinhyun.Kim@utdallas.edu (J.H.K.); antonio.lucero@qorvo.com (A.T.L.); arul.ravichandran@asm.com (A.R.); Harrison.Kim@utdallas.edu (H.S.K.); 2 Department of Materials Science and Engineering, Inha University, 100 Inha-ro, Michuhol-gu, Incheon 22212, Korea; Rino.Choi@inha.ac.kr; 3 Department of Electrical and Electronics Engineering, Kangwon National University, 1 Gangwondaehakgil, Chuncheon, Gangwon-do 24341, Korea; sijoon.kim@kangwon.ac.kr; 4 Division of Materials Science and Engineering, Hanyang University, 222 Wangsimni-ro, Seongdong-gu, Seoul 04763, Korea; jhahn@hanyang.ac.kr; 5 RASIRC Inc., 7815 Silverton Avenue, San Diego, CA 92126, USA; dalvarez@rasirc.com (D.A.); js@rasirc.com (J.S.)

**Keywords:** atomic layer deposition (ALD), aluminum nitride, hydrazine, trimethyl aluminum (TMA)

## Abstract

Aluminum nitride (AlN) thin films were grown using thermal atomic layer deposition in the temperature range of 175–350 °C. The thin films were deposited using trimethyl aluminum (TMA) and hydrazine (N_2_H_4_) as a metal precursor and nitrogen source, respectively. Highly reactive N_2_H_4_, compared to its conventionally used counterpart, ammonia (NH_3_), provides a higher growth per cycle (GPC), which is approximately 2.3 times higher at a deposition temperature of 300 °C and, also exhibits a low impurity concentration in as-deposited films. Low temperature AlN films deposited at 225 °C with a capping layer had an Al to N composition ratio of 1:1.1, a close to ideal composition ratio, with a low oxygen content (7.5%) while exhibiting a GPC of 0.16 nm/cycle. We suggest that N_2_H_4_ as a replacement for NH_3_ is a good alternative due to its stringent thermal budget.

## 1. Introduction

Aluminum nitride (AlN) is one of the promising materials for electronic and optoelectronic devices due to its wide band gap structure (6.2 eV), high thermal conductivity (2.85 W/cm·K at 300 K), melting point (2750 °C), and large critical electric field (12 MV/cm) [1,2,3]. Additionally, using a highly thermal conductive material like AlN as a thermal spreader can result in enhanced thermal dissipation, which is highly beneficial in scaled devices [4,5,6]. These nitride deposition processes should be compatible with the thermal budget of back-end-of-line (BEOL) processes in conventional complementary metal-oxide-semiconductor (CMOS) fabrication. A lower deposition temperature (<300 °C) is preferred and conformality over high-aspect ratio structures, commonly found in novel, complex device structures, is also desirable. Hence, the atomic layer deposition (ALD) technique is a forerunner among other deposition techniques that meets these specifications while providing excellent thickness controllability. Plasma-enhanced ALD (PEALD) provides the plasma radicals required to push the boundaries of ALD reactions towards a lower temperature but sensitive substrates can suffer from plasma-induced damage [7]. PEALD has a relatively poor conformal deposition on complicated 3D nano-structures compared to thermal ALD.

**Figure 1:**
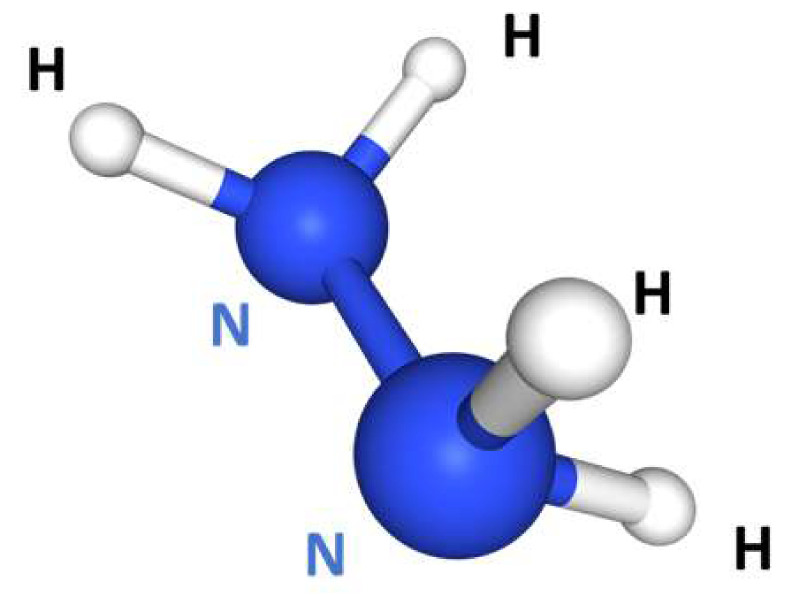
Ball stick model of hydrazine (N_2_H_4_) with N atoms and H atoms.

Currently, AlN ALD using ammonia (NH_3_) and trimethyl aluminum (TMA) results in an incomplete reaction at temperatures below 300 °C [8]. High-temperature ALD above 450 °C is required in order to achieve a vigorous reaction with methyl groups (–CH_3_) and the complete removal of by-products in the ALD process using NH_3_ and TMA [8]. One way of circumventing this issue is by introducing a more reactive nitrogen source than NH_3_. From this perspective, hydrazine (N_2_H_4_) can be used as a replacement for NH_3_ as the N–N bond in N_2_H_4_ (~167 kJ/mol) is weak when compared to the N–H bond (~386 kJ/mol) in NH_3_ [9]. The molecular structure of N_2_H_4_ is as shown in Figure 1 using a ball-stick model. Safety was a key concern while handling N_2_H_4_, but the newly available ultra-high purity anhydrous N_2_H_4_ source is compliant with the safety standard requirements and has also demonstrated the deposition of metal nitrides at low temperatures [10,11,12].

Abdulagatov et al. recently demonstrated AlN deposition by thermal ALD using tris(diethylamido)aluminum (III) (TDEAA) and hydrazine in the deposition temperature range from 150 to 280 °C [13]. Growth rates of 1.23, 1.16, and 1.72 Å/cycle were reported at 150, 200, and 280 °C, respectively. The higher growth rate observed at 280 °C was mainly attributed to the organo-metallic precursor decomposition and hence a chemical vapor deposition (CVD) reaction mechanism was suspected for observing such a high growth rate. Additionally, it was also demonstrated that the impurity, such as carbon and oxygen content, in nitride film deposited using hydrazine was comparable or lower when compared to films deposited using NH_3_ [13,14].

Previous studies revealed that the growth rate of AlN is less than 0.04 nm/cycle by thermal ALD at temperatures below 400 °C using TMA and NH_3_ [13]. Furthermore, TMA starts to decompose at higher temperatures (above 377 °C) [15] and reduces the film quality of AlN [16,17]. In order to deposit high-quality AlN with a reasonable growth rate at low temperatures, it is essential to adopt highly reactive precursors, such as hydrazine, into the ALD process. In this paper, we successfully demonstrate AlN films deposition by thermal ALD at low temperatures. Ultra-pure anhydrous N_2_H_4_ and TMA were used to deposit AlN thin films in the temperature range from 175 to 350 °C, with the feasibility of AlN deposition using an Al ALD precursor of TMA. As a comparison, the growth rate and surface roughness of AlN films deposited by thermal ALD using TMA and NH_3_ are also presented.

## 2. Materials and Methods

### 2.1. Film Deposition

**Figure 2:**
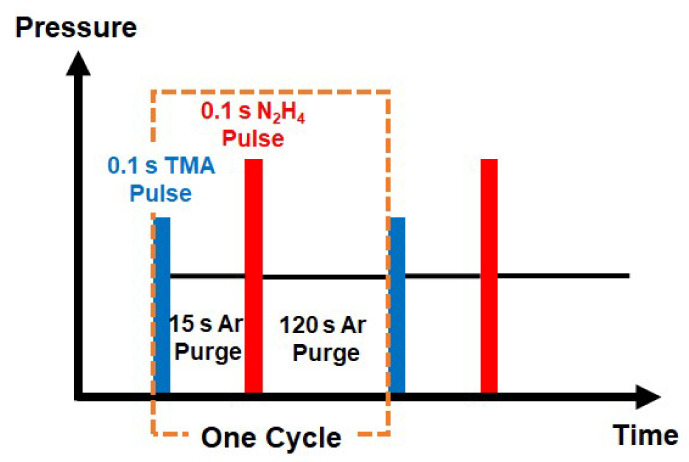
Schematic of atomic layer deposition (ALD) cycle for deposition of AlN using trimethylaluminum (TMA) and hydrazine (N_2_H_4_). One cycle of the ALD process condition is: TMA pulse (0.1 s)–Ar purge (15 s)–N_2_H_4_ pulse (0.1 s)–Ar purge (120 s).

ALD AlN was deposited using TMA and N_2_H_4_ as the Al precursor and nitrogen source, respectively. The films were deposited using home-built ALD system with a hollow-cathode plasma source (Meaglow Ltd., Thunder Bay, Canada) provision to generate plasma. This ALD system has been used for deposition of various nitride films using thermal ALD or PEALD processes [18,19,20,21]. The stainless-steel chamber wall was heated to ~120 °C and the precursor delivery lines were maintained at 90 °C to avoid condensation. The precursors were maintained at room temperature. For the ALD process, p-type Si (100) substrates (Silicon Valley Microelectronics, Santa Clara, CA, USA) with a resistivity of 3–10 Ω·cm were dipped in 100:1 diluted HF solution to remove native oxide. After blowing with N_2_, the substrates were directly transferred to the process chamber. During this ex situ process, re-oxidation of the silicon surface was negligible to cause interdiffusion of oxygen into AlN films [22]. After loading the substrates, the chamber was pumped down to 10^−6^ Torr using a turbomolecular pump to reduce adventitious contaminants introduced during the substrate transfer. The process pressure was maintained at 0.5 Torr with continuous flow of Ar carrier gas. The representative time sequence of one cycle of the ALD process condition was set to be: TMA pulse (0.1 s)–Ar purge (15 s)–N_2_H_4_ pulse (0.1 s)–Ar purge (120 s), as shown in Figure 2. The deposition temperature was varied between 175 and 350 °C. For comparison, all samples in this study were deposited using 100 cycles.

In the case of material characterization of AlN grown at 225 °C, 4 nm-thick silicon nitride (SiN_x_) was deposited as a capping layer in order to prevent the surface oxidation in air. The SiN_x_ capping layer was subsequently deposited at 410 °C using hexachlorodisilane (Si_2_Cl_6_) and N_2_H_4_ in the same chamber without breaking the vacuum.

### 2.2. Film Characterization

The thickness and refractive index (R.I.) of AlN thin films were measured by spectroscopic ellipsometry (SE, M-2000DI, J.A. Woolam, Lincoln, NE, USA) and the values were fit using the spectra measured at 3 different angles (55°, 65°, and 75°). The chemical composition and bonding states of AlN thin films were characterized by X-ray photoelectron spectroscopy (XPS). XPS analysis was performed using a PHI VersaProbe II (ULVAC-PHI, Chigasaki, Kanagawa, Japan) equipped with a monochromatic Al Kα X-ray source (E_Photon_ = 1486.6 eV). To remove surface contaminants, Ar gas cluster ion beam (GCIB) sputtering with a beam energy of 1 kV and the cluster size of 2500 atoms was employed. The elemental composition of the films was calculated based on the peak area and atomic sensitivity factor [23]. Surface roughness of AlN films was determined by atomic force microscope (AFM) (Veeco Multimode V. Non-contact AFM, Veeco, Plainview, NY, USA).

## 3. Results and Discussion

**Figure 3:**
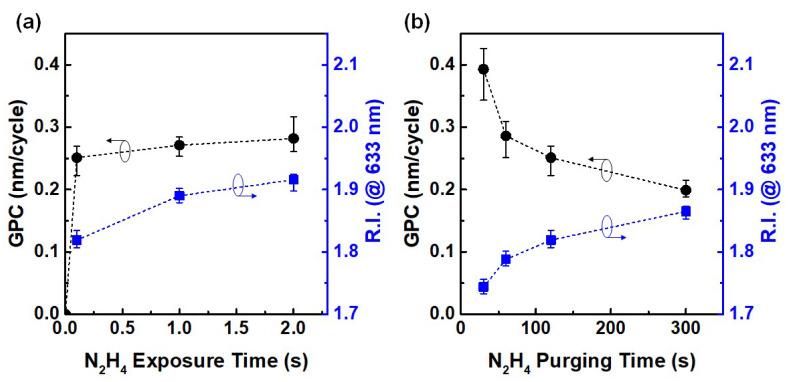
Saturation curves for the growth-per-cycle (GPC) for (**a**) hydrazine pulse and (**b**) purge time. The ALD process was performed at a substrate temperature of 300 °C. The refractive index (R.I.) at 633 nm of the deposited films was measured using spectroscopic ellipsometer after deposition.

The ALD deposition of AlN using TMA and N_2_H_4_ followed by sequentially pulsing the reactants, followed by flushing out the reaction by-products using Ar between the self-limited surface reactions. To get the ALD process condition, self-limited growth per cycle (GPC) characteristics were examined by increasing N_2_H_4_ pulse and purge time, as shown in Figure 3a,b. With the increased N_2_H_4_ pulse and purge time, the growth rate saturated at a constant level as expected in the ideal ALD process. An increase of purging time (300 s or longer) might be inappropriate for the ALD process due to the feasibility, hence we set the pulse and purge time of N_2_H_4_ for AlN deposition process to 0.1 s and 120 s, respectively. Meanwhile, the R.I. of film at wavelength of 633 nm was slightly larger (<0.1) as the pulse and purge time increased. This variation was not sufficient to argue that the stoichiometry of the deposited films has changed. In addition, it was confirmed that the AlN thin films deposited by the thermal ALD process had an amorphous nature, as confirmed by XRD analysis. The density of the thin films deposited at 350 °C confirmed through X-ray reflectometry analysis was 2.9 g/cm^3^, which is about 10% lower value compared to the reference value of bulk crystalline AlN of 3.26 g/cm^3^. The unit cell dimensions of the hexagonal structure of wurtzite AlN were reported as a = 3.1151 Å, b = 3.1151 Å, and c = 4.9880 Å [24]. Therefore, a relatively larger GPC is expected than when the crystalline AlN is grown.

**Figure 4:**
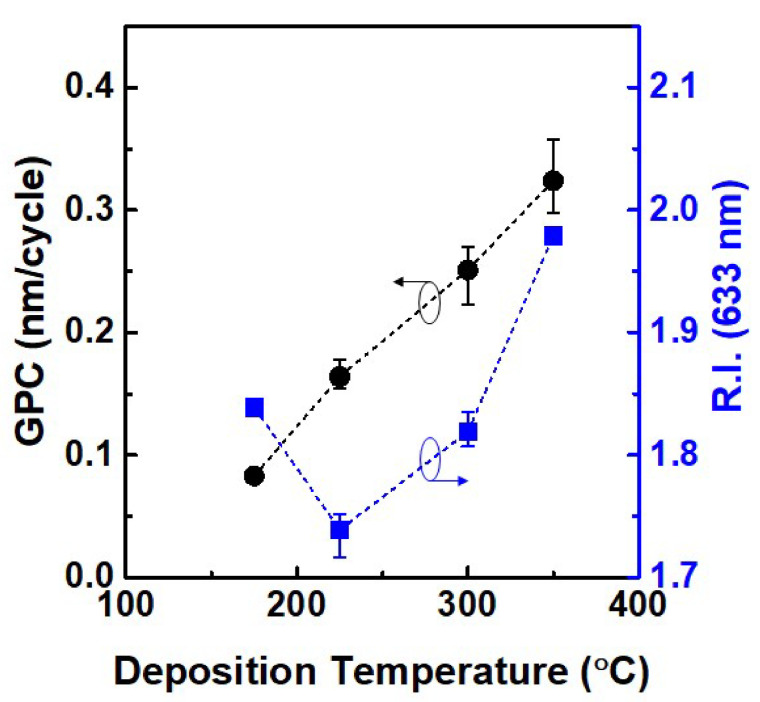
Growth-per-cycle (GPC) of AlN thin films as a function of the deposition temperature and refractive index (R.I.) at the wavelength of 644 nm with different deposition temperatures for trimethylaluminum (TMA) and hydrazine (N_2_H_4_) precursors.

Figure 4 shows the temperature dependence of the film growth rate from 175 to 350 °C. To obtain the GPC of each point in Figure 4, the AlN films were deposited using 100 ALD cycles. The growth rate increased linearly with increasing deposition temperature, where the GPC was 0.08, 0.16, 0.25, and 0.32 nm/cycle at 175, 225, 300 and 350 °C, respectively. In an ideal ALD process, the constant GPC can be achieved at temperatures high enough to avoid precursor condensation and satisfy perceptible reactivity between the precursor and substrate, but sufficiently low to prevent precursor decomposition and desorption of chemisorbed species from the surface [25,26]. Nevertheless, the GPC can still be varied with temperature while maintaining self-limiting growth due to the temperature dependence of the reactive sites on the surface and the reaction mechanism of the precursor itself [26]. Our observation confirmed that co-adsorption of TMA and N_2_H_4_ was self-limiting by forming a monolayer at substrate temperature <350 °C, while above this temperature reaction was rapid and formed a thick film of AIN. Consequently, the CVD effect became significant and an increase of GPC could be observed [16,17] as deposition temperature is up to 350 °C. It is also worth noting that the ALD window of AlN using TMA is narrow in earlier studies [27,28]. Furthermore, the growth rate of thermal ALD-AlN films from TMA and NH_3_ was inconsistent in previous reports. For example, Tian et al. reported the growth rate of AlN films was 0.01 nm/cycle at 375 °C [27], while Kim et al. deposited AlN films in the temperature range from 265 to 335 °C with a growth rate of 0.02–0.16 nm/cycle [29]. Unfortunately, in the case of AlN deposited using NH_3_ in a thermal ALD process, the sub-angstrom (less than 0.5 Å) growth rate was too low for accurate comparison. On the other hand, the deposition rates observed in our study using N_2_H_4_ were much larger than those reported earlier for thermal ALD AlN using TMA and NH_3_. To suppress changes from equipment difference, we deposited AlN using NH_3_ as the nitrogen source in the same ALD reactor. As a result, it was confirmed that the deposition rates at 300 °C increased by 2.3 times with N_2_H_4_.

The R.I. of the AlN film at wavelength of 633 nm was extracted from the SE. The SE data were fit using the Cauchy model, which is widely used for semiconductor materials [30]. The R.I. of the AlN film increases with increasing deposition temperature except at 175 °C. It is suspected that the higher R.I. at 175 °C can be attributed by hydrogen species inside the film. Due to the relatively low temperature, the remaining N–H bonds from N_2_H_4_ are dominant after ligand exchange with TMA, resulting in the lower GPC results [31]. The R.I. of AlN film deposited at 350 °C is 1.98, which is close to the reported values of high-quality AlN films [32,33,34].

**Figure 5:**
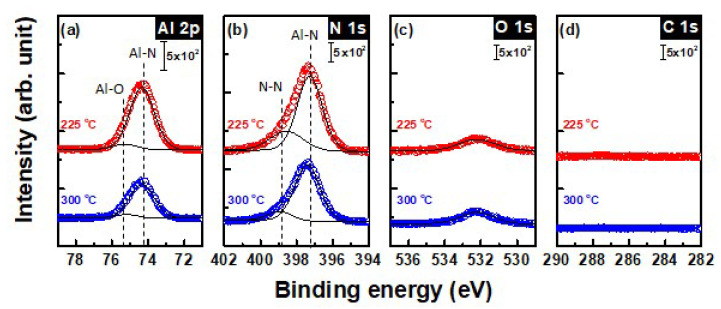
High resolution X-ray photoelectron spectroscopy (XPS) (**a**) Al 2p, (**b**) N 1s, (**c**) O 1s, and (**d**) C 1s spectra of AlN thin film deposited at 225 and 300 °C.

XPS measurements were performed to investigate chemical bonding states of AlN thin films. Figure 5 shows XPS analysis results of AlN films deposited for 100 ALD cycles at 225 and 300 °C. AlN films, when exposed to air, react with oxygen and water to form an aluminum oxide film [34]. As mentioned in the experimental section, we deposited SiN_x_ on AlN film grown at 225 °C to prevent the ambient oxidation of the nitride film deposited below 225 °C. It was an inevitable choice to passivate with the SiN_x_ film, because ex-situ chemical analysis was impossible owing to the oxidation being too active in the air. The O 1s peak for the AlN films deposited at 225 °C with and without capping layer is depicted in Supplementary Materials (Figure S1). It showed that 4 nm-thick SiN_x_ capping layer provides effective barrier to oxidation of AlN surface. Nevertheless, it was indicated that the peak at 532.4 eV is assigned to Al–O bonds in the AlN films, and these oxygen impurities in both films were considered the natural characteristics of the AlN films [35,36,37]. It is worth noting that the capping layer should be thin enough to avoid significant signal attenuation from the sample and there should be no interfacial reaction with the sample [38,39,40]. Meanwhile AlN films deposited at 300 °C do not have any capping layer, which may result in higher O content in the films compared to the films deposited at 225 °C with a capping layer. It should be noted that the peak positions from the AlN film with the capping layer were calibrated using the Si 2p peak at 99.4 eV, which comes from the Si substrate. In the case of AlN films without capping, the peak positions were calibrated with Al 2p peak at 74.3 eV, the calibrated peaks from the AlN films aforementioned. All narrow scans were deconvoluted for more accurate analysis of the chemical bonding status, such as metal oxide and metal nitrides. Both the Al 2p and N 1s peaks were slightly asymmetric, indicating the presence of different bonding features associated with nitrogen in the AlN thin films.

Figure 5a shows the comparison of the Al 2p peaks for the AlN films deposited at 225 and 300 °C. Deconvolution of the Al 2p spectrum gives rise to two peaks, one at 74.3 eV, which corresponds to Al in Al–N, and the other at 75.2 eV, which corresponds to the BE of Al–O bonds. The values of these peaks are comparable with the previously reported spectra for AlN films [28,41,42]. As shown in Figure 5a, mainly Al–N bonds and ignorable Al–O bonds were observed. In the same way, the N 1s spectrum was deconvoluted with two peaks as described in Figure 5b. It showed one main peak centered at a BE of 397.4 eV, corresponds to N in Al–N, and the other at 398.6 eV, corresponds to the BE of unbounded nitrogen [43].

There is a concern regarding the source of unbounded nitrogen observed in the film. We suggest that the unbounded nitrogen came from incompletely reacted N_2_H_4_ without breaking the N–N bond, which remains after reaction with TMA due to low deposition temperature. This hypothesis is also supported by the decrease in GPC with increase in N_2_H_4_ purge time, as shown in Figure 3b. The composition distribution of the AlN films was also analyzed. This elemental analysis for the AlN films is described in Table 1 which shows that the surface composition consists of aluminum, nitrogen and oxygen, as expected. The surface composition analysis of sputtered film requires attention to interpret due to the preferential Ar ion sputtering effect [42]. Due to the unbroken N–N bonds of N_2_H_4_, it is supposed that the Al:N ratio is 1:1; however, the ratio increased over 1:1 with higher nitrogen concentration. The total [N]/[Al] ratio of the AlN film deposited at 225 and 300 °C was 1.1 and 1.2, respectively. Although this difference is small, N 1s spectra clearly indicates the AlN film deposited at 225 °C shows higher unbounded nitrogen content. The decrement of [N]/[N–Al] ratio from 0.34 to 0.15 with increasing deposition temperature may suggest that the films deposited at higher deposition temperature have larger R.I., i.e., high density. Nevertheless, the AlN thin film deposited at 225 °C showed an Al:N ratio of 1:1.1, which meant that stoichiometric AlN was successfully deposited.

**Figure 6:**
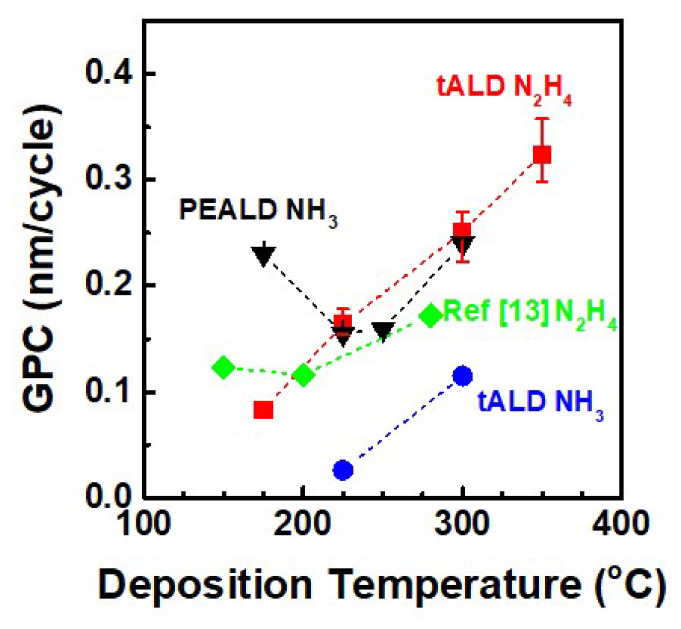
Comparison of growth per cycle (GPC) of AlN thin films deposited using TMA and hydrazine (N_2_H_4_) and ammonia (NH_3_), as the metal precursor and nitrogen source, respectively. The AlN films were deposited by using thermal atomic layer deposition (tALD) and plasma-enhanced atomic layer deposition (PEALD) technique. It is also indicated that GPC of AlN thin films deposited using TDEAA and N_2_H_4_, as the metal precursor and nitrogen source [13].

For more precise comparison of the growth rate, we conducted ALD of AlN using different techniques, using the same ALD reactor. As shown in Figure 6, the growth rates of tALD N_2_H_4_, PEALD NH_3_, and tALD NH_3_ were 0.16, 0.15, and 0.03 nm/cycle at 225 °C and 0.25, 0.24, and 0.11 nm/cycle at 300 °C, respectively. When using N_2_H_4_ as the nitrogen source, the growth rates were 5.3 and 2.3 times higher than when deposited by tALD using NH_3_ at 225 and 300 °C, respectively. In addition, the growth rate of AlN deposited by tALD N_2_H_4_ was comparable with that of AlN deposited by PEALD NH_3_.

**Figure 7:**
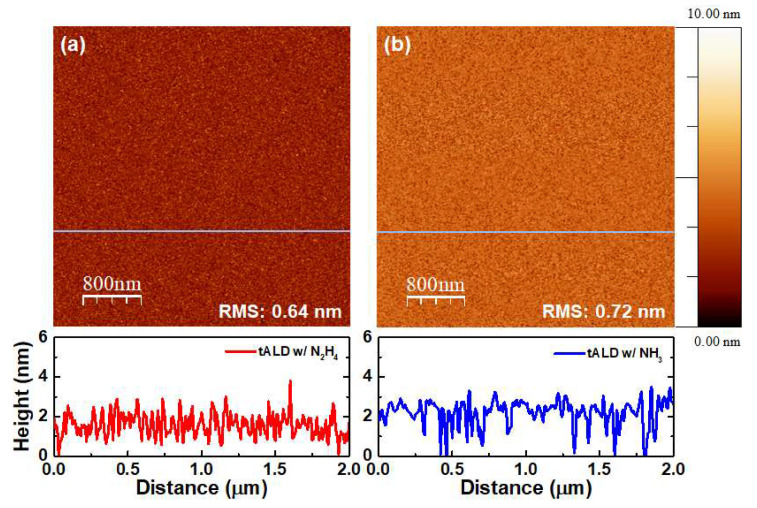
Surface roughness of AlN thin films deposited using (**a**) hydrazine (N_2_H_4_) and (**b**) ammonia (NH_3_), as the nitrogen source. Root-mean-square (RMS) values of AlN thin films deposited using N_2_H_4_ and NH_3_ were 0.64 and 0.72 nm, respectively.

Figure 7 shows the surface morphology of AlN thin films grown by thermal ALD using two different nitrogen sources, N_2_H_4_ and NH_3_. The root-mean-square (RMS) roughness of the films were measured by AFM and their values were 0.64 and 0.72 nm for AlN films deposited using 100 cycles, using N_2_H_4_ and NH_3_ as the nitrogen source, respectively. There is no degradation of surface roughness with N_2_H_4_ despite the higher growth rate, which makes N_2_H_4_ an attractive nitrogen source in semiconductor fabrication.

## 4. Conclusions

Deposition of low-temperature AlN through thermal ALD has been demonstrated. Optical and chemical characterization has been performed to get an accurate assessment of the quality of the deposited AlN films. Thicknesses and R.I.s of the films were analyzed using SE. A growth rate of 0.16 nm/cycle and R.I. of 1.74 was obtained at the very low temperature of 225 °C. A high growth rate can be achieved compared to NH_3_ as the nitrogen source due to the high reactivity of N_2_H_4_. XPS results showed an Al:N ratio of 1:1.1 and few impurities in the films.

In summary, stable AlN thin films were successfully grown while the material parameters were comparable to those of aluminum nitride films deposited using NH_3_. In addition, the rapid deposition rate at low temperatures demonstrates the potential of the nitrogen source to replace NH_3_.

## Figures and Tables

**Table 1:** Chemical composition of the AlN films deposited at 225 and 300 °C as determined by high resolution XPS analysis.

Deposition Temperature	[Al] at.%	[N] at.%	[O] at.%	[C] at.%	[N]/[Al]	[N]/[N–Al]
225 °C	44.8	47.7	7.5	<d.l.^1^	1.1	0.34
300 °C	40.6	49.4	10.0	<d.l.^1^	1.2	0.15

^1^ Detection limit (d.l.).

## Supplementary Materials

The following are available online at https://www.mdpi.com/1996-1944/13/15/3387/s1, Figure S1: High resolution O 1s XPS spectra of (**a**) AlN deposited at 225 °C w/ and w/o capping layer and (**b**) AlN deposited at 225 °C w/ capping layer and deposited at 300 °C w/o capping layer.

## References

[1] Slack G.A., Tanzilli R.A., Pohl R.O., Vandersande J.W. (1987). The Intrinsic Thermal Conductivity of AIN. J. Phys. Chem. Solids.

[2] Zhang Z., Gao B., Fang Z., Wang X., Tang Y., Sohn J., Wong H.S.P., Wong S.S., Lo G.Q. (2015). All-Metal-Nitride RRAM Devices. IEEE Electron Device Lett..

[3] Xiong C., Pernice W.H.P., Sun X., Schuck C., Fong K.Y., Tang H.X. (2012). Aluminum Nitride as a New Material for Chip-Scale Optomechanics and Nonlinear Optics. New J. Phys..

[4] Kuo P.K., Auner G.W., Wu Z.L. (1994). Microstructure and thermal conductivity of epitaxial AlN thin films. Thin Solid Films.

[5] Park M.-H., Kim S.-H. (2012). Thermal conductivity of AlN thin films deposited by RF magnetron sputtering. Mater. Sci. Semicond. Process..

[6] Xu R.L., Rojo M.M., Islam S.M., Sood A., Vareskic B., Katre A., Mingo N., Goodson K.E., Xing H.G., Jena D. (2019). Thermal conductivity of crystalline AlN and the influence of atomic-scale defects. J. Appl. Phys..

[7] Knoops H.C.M., Faraz T., Arts K., Kessels W.M.M. (2019). Status and Prospects of Plasma-Assisted Atomic Layer Deposition. J. Vac. Sci. Technol. A.

[8] Puurunen R.L., Lindblad M., Rootc A., Krausea A.O.I. (2001). Successive Reactions of Gaseous Trimethylaluminium and Ammonia on Porous Alumina. Phys. Chem. Chem. Phys..

[9] Huheey J.E. (1983). Inorganic Chemistry.

[10] Alvarez D., Spiegelman J., Andachi K., Holmes R., Raynor M., Shimizu H. Enabling Low Temperature Metal Nitride ALD Using Ultra-High Purity Hydrazine: ET/ID: Enabling Technologies and Innovative Devices. Proceedings of the 2017 28th Annual SEMI Advanced Semiconductor Manufacturing Conference.

[11] Wolf S., Edmonds M., Sardashti K., Clemons M., Park J.H., Yoshida N., Dong L., Nemani S., Yieh E., Holmes R. (2018). Low-Temperature Amorphous Boron Nitride on Si_0.7_Ge_0.3_(001), Cu, and HOPG from Sequential Exposures of N_2_H_4_ and BCl_3_. Appl. Surf. Sci..

[12] Hwang S.M., Pena L.F., Kim H.S., Kondusamy A.L.N., Qin Z., Jung Y.C., Veyan J.-F., Alvarez D., Spiegelman J., Kim J. (2019). Vapor-phase Surface Cleaning of Electroplated Cu Films Using Anhydrous N_2_H_4_. ECS Trans..

[13] Abdulagatov A.I., Ramazanov S.M., Dallaev R.S., Murliev E.K., Palchaev D.K., Rabadanov M.K., Abdulagatov I.M. (2018). Atomic Layer Deposition of Aluminum Nitride Using Tris(Diethylamido)Aluminum and Hydrazine or Ammonia. Russ. Microelectron..

[14] Abdulagatov A.I., Amashaev R.R., Ashurbekova K.N., Ashurbekova K.N., Rabadanov M.K., Abdulagatov I.M. (2018). Atomic Layer Deposition of Aluminum Nitride and Oxynitride on Silicon Using Tris(dimethylamido)aluminum, Ammonia, and Water. Russ. J. Gen. Chem..

[15] Mayer T.M., Rogers J.W., Michalske T.A. (1991). Mechanism of Nucleation and Atomic Layer Growth of AlN on Si. Chem. Mater..

[16] Liu S., Peng M., Hou C., He Y., Li M., Zheng X. (2017). PEALD-Grown Crystalline AlN Films on Si(100) with Sharp Interface and Good Uniformity. Nanoscale Res. Lett..

[17] Riihela D., Ritala M., Matero R., Leskela M., Jokinen J., Haussalo P. (1996). Low Temperature Deposition of AlN Films by an Alternate Supply of Trimethyl Aluminum and Ammonia. Chem. Vap. Depos..

[18] Meng X., Kim H.S., Lucero A.T., Hwang S.M., Lee J.S., Byun Y.C., Kim J., Hwang B.K., Zhou X., Young C. (2018). Hollow Cathode Plasma-Enhanced Atomic Layer Deposition of Silicon Nitride Using Pentachlorodisilane. ACS Appl. Mater. Interfaces.

[19] Meng X., Lee J., Ravichandran A., Byun Y.C., Lee J.G., Lucero A.T., Kim S.J., Ha M.W., Young C.D., Kim J. (2018). Robust SiN_x_/GaN MIS-HEMTs With Crystalline Interfacial Layer Using Hollow Cathode PEALD. IEEE Electron Device Lett..

[20] Kim H.S., Meng X., Kim S.J., Lucero A.T., Cheng L., Byun Y.-C., Lee J.S., Hwang S.M., Kondusamy A.L.N., Wallace R.M. (2018). Investigation of the Physical Properties of Plasma Enhanced Atomic Layer Deposited Silicon Nitride as Etch Stopper. ACS Appl. Mater. Interfaces.

[21] Hwang S.M., Kondusamy A.L.N., Qin Z., Kim H.S., Meng X., Kim J., Hwang B.K., Zhou X., Telgenhoff M., Young J. (2019). Hollow Cathode Plasma (HCP) Enhanced Atomic Layer Deposition of Silicon Nitride (SiN_x_) Thin Films Using Pentachlorodisilane (PCDS). ECS Trans..

[22] Zazzera L.A., Moulder J.F. (1989). XPS and SIMS Study of Anhydrous HF and UV/Ozone-Modified Silicon (100) Surfaces. J. Electrochem. Soc..

[23] Moulder J.F., Stickle W.F., Sobol P.E., Bomben K.D. (1992). Handbook of X-Ray Photoelectron Spectroscopy.

[24] Paterson W.G., Onyszhuk M. (1961). Co-ordination compounds of hydrazine Part 2.:The Interaction of Trimethylborane and Trimethylaluminum with Hydrazine. Can. J. Chem..

[25] George S.M. (2010). Atomic layer deposition: An overview. Chem. Rev..

[26] Sønsteby H.H., Yanguas-Gil A., Elam J.W. (2020). Consistency and Reproducibility in Atomic Layer Deposition. J. Vac. Sci. Technol. A.

[27] Tian L., Ponton S., Benz M., Crisci A., Reboud R., Giusti G., Volpi F., Rapenne L., Vallee C., Pons M. (2018). Aluminum Nitride Thin Films Deposited by Hydrogen Plasma Enhanced and Thermal Atomic Layer Deposition. Surf. Coat. Technol..

[28] Banerjee S., Aarnink A.A., Kruijs R., Kovalgin A.Y., Schmitz J. (2015). PEALD AlN: Controlling growth and film crystallinity. Phys. Status Solidi C.

[29] Kim Y., Kim M.S., Yun H.J., Ryu S.Y., Choi B.J. (2018). Effect of Growth Temperature on AlN Thin Films Fabricated by Atomic Layer Deposition. Ceram. Int..

[30] Khoshman J.M., Kordesch M.E. (2005). Spectroscopic ellipsometry characterization of amorphous aluminum nitride and indium nitride thin films. Phys. Status Solidi C.

[31] Tzou A.-J., Chu K.-H., Lin I.-F., Østreng E., Fang Y.-S., Wu X.-P., Wu B.-W., Shen C.-H., Shieh J.-M., Yeh W.-K. (2017). AlN Surface Passivation of GaN-Based High Electron Mobility Transistors by Plasma-Enhanced Atomic Layer Deposition. Nanoscale Res. Lett..

[32] Shih H.-Y., Lee W.-H., Kao W.-C., Chuang Y.-C., Lin R.-M., Lin H.-C., Shiojiri M., Chen M.-J. (2018). Low-temperature Atomic Layer Epitaxy of AlN Ultrathin Films by Layer-by-Layer, in-situ Atomic Layer Annealing. Sci. Rep..

[33] Brunner D. (1997). Optical Constants of Epitaxial AlGaN Films and Their Temperature Dependence. J. Appl. Phys..

[34] Özgür Ü., Webb-Wood G., Everitt H.O., Yun F., Morkoç H. (2001). Systematic Measurement of Al_x_Ga_1−x_N Refractive Indices. Appl. Phys. Lett..

[35] Ozgit-Akgun C., Goldenberg E., Okyay A.K., Biyikli N. (2014). Hollow Cathode Plasma-assisted Atomic Layer Deposition of Crystalline AlN, GaN and Al_x_Ga_1−x_N Thin Films at Low Temperatures. J. Mater. Chem. C.

[36] Rosenberger L., Baird R., McCullen E., Auner G., Shreve G. (2008). XPS Analysis of Aluminum Nitride Films Deposited by Plasma Source Molecular Beam Epitaxy. Surf. Interface Anal..

[37] Zhang J., Zhang Q., Yang H., Wu H., Zhou J., Hu L. (2014). Bipolar Resistive Switching Properties of AlN Films Deposited by Plasma-enhanced Atomic Layer Deposition. Appl. Surf. Sci..

[38] Greczynski G., Petrov I., Greene J.E., Hultman L. (2015). Al Capping Layers for Nondestructive X-ray Photoelectron Spectroscopy Analyses of Transition-metal Nitride Thin Films. J. Vac. Sci. Technol. A.

[39] Greczynski G., Primetzhofer D., Lu J., Hultman L. (2017). Core-level Spectra and Binding Energies of Transition Metal Nitrides by Non-destructive X-ray Photoelectron Spectroscopy Through Capping Layers. Appl. Surf. Sci..

[40] Muneshwar T., Cadien K. (2018). Comparing XPS on Bare and Capped ZrN films Grown by Plasma Enhanced ALD: Effect of Ambient Oxidation. Appl. Surf. Sci..

[41] Jokinen J., Haussalo P., Keinonen J., Ritala M., Riihela D., Leskela M. (1996). Analysis of AlN Thin Films by Combining TOF-ERDA and NRB Techniques. Thin Solid Films.

[42] Motamedi P., Cadien K. (2014). XPS Analysis of AlN Thin Films Deposited by Plasma Enhanced Atomic Layer Deposition. Appl. Surf. Sci..

[43] Taborda J.A.P., Landazuri H.R., Londono L.P.V. (2016). Correlation Between Optical, Morphological, and Compositional Properties of Aluminum Nitride Thin Films by Pulsed Laser Deposition. IEEE Sens. J..

