# Supplementary material for: Low Temperature Thermal Atomic Layer Deposition of Aluminum Nitride Using Hydrazine as the Nitrogen Source
Source: Materials (Basel). 2020 Jul 31;13(15):3387. doi: 10.3390/ma13153387 (PMC7436040; doi:10.3390/ma13153387)
Supplement: Supplementary file 1 [file materials-13-03387-s001.pdf]

materials

Supplementary Materials

Low Temperature Thermal Atomic Layer Deposition of Aluminum Nitride Using Hydrazine as the Nitrogen Source

Yong Chan Jung, Su Min Hwang, Dan N. Le, Aswin L. N. Kondusamy, Jaidah Mohan, Sang Woo Kim, Jin Hyun Kim, Antonio T. Lucero, Arul Ravichandran, Harrison Sejoon Kim, Si Joon Kim, Rino Choi, Jinho Ahn, Daniel Alvarez, Jeff Spiegelman and Jiyoung Kim

In the case of AlN film deposited at 225 °C, we noticed a high amount of oxygen impurities (more than 30 at.%) after 4 min of Ar sputtering. Therefore, we deposited a 4 nm-thick SiN_x capping layer after AlN film was grown at 225 °C without breaking the vacuum (in the same reactor) for XPS analysis. Figure S1a shows that the SiN_x capping layer provided effective barriers to oxidation of AlN surfaces. As shown in Figure S1b, for the deposition done at 300 °C, the oxidation was comparable to deposition at 225 °C with a capping layer. Hence, there was no need to deposit a capping layer.

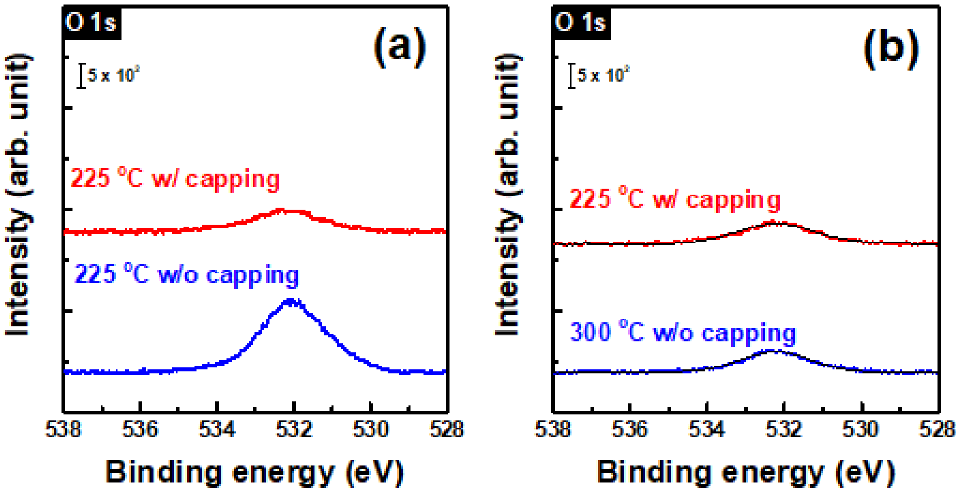

Figure S1. High resolution O 1s XPS spectra of (**a**) AlN deposited at 225 °C w/ and w/o capping layer and (**b**) AlN deposited at 225 °C w/ capping layer and deposited at 300 °C w/o capping layer.

© 2020 by the authors. Licensee MDPI, Basel, Switzerland. This article is an open access article distributed under the terms and conditions of the Creative Commons Attribution (CC BY) license (http://creativecommons.org/licenses/by/4.0/).
